# Making the Switch: Alternatives to Fetal Bovine Serum for Adipose-Derived Stromal Cell Expansion

**DOI:** 10.3389/fcell.2016.00115

**Published:** 2016-10-17

**Authors:** Carla Dessels, Marnie Potgieter, Michael S. Pepper

**Affiliations:** South African Medical Research Council, Extramural Unit for Stem Cell Research and Therapy, and Department of Immunology, Faculty of Health Sciences, Institute for Cellular and Molecular Medicine, University of PretoriaPretoria, South Africa

**Keywords:** adipose-derived stromal cells, fetal bovine serum, good manufacturing processes, *in vitro* expansion, human serum, platelet rich plasma, platelet poor plasma, platelet lysate

## Abstract

Adipose-derived stromal cells (ASCs) are being used extensively in clinical trials. These trials require that ASCs are prepared using good manufacturing practices (GMPs) and are safe for use in humans. The majority of clinical trials in which ASCs are expanded make use of fetal bovine serum (FBS). While FBS is used traditionally in the research setting for *in vitro* expansion, it does carry the risk of xenoimmunization and zoonotic transmission when used for expanding cells destined for therapeutic purposes. In order to ensure a GMP quality product for cellular therapy, *in vitro* expansion of ASCs has been undertaken using xeno-free (XF), chemically-defined, and human blood-derived alternatives. These investigations usually include the criteria proposed by the International Society of Cellular Therapy (ISCT) and International Fat Applied Technology Society (IFATS). The majority of studies use these criteria to compare plastic-adherence, morphology, the immunophenotype and the trilineage differentiation of ASCs under the different medium supplemented conditions. Based on these studies, all of the alternatives to FBS seem to be suitable replacements; however, each has its own advantages and drawbacks. Very few studies have investigated the effects of the supplements on the immunomodulation of ASCs; the transcriptome, proteome and secretome; and the ultimate effects in appropriate animal models. The selection of medium supplementation will depend on the downstream application of the ASCs and their efficacy and safety in preclinical studies.

## Introduction

Adipose-derived stromal cells (ASCs) are multipotent and immunoprivileged, making them ideal candidates for therapeutic purposes (Bourin et al., 2013; Ma et al., 2014; Kallmeyer and Pepper, 2015). ASCs can be isolated using minimally invasive techniques from various adipose tissue depots in the body (Zuk et al., 2001). They are characterized by their ability to adhere to plastic, a unique surface marker profile and the capacity to differentiate into bone, fat and cartilage (Dominici et al., 2006; Bourin et al., 2013). ASCs comprise ~15–30% of the stromal vascular fraction (SVF) of adipose tissue (Bourin et al., 2013; Zuk, 2013), and need to be expanded *ex vivo* in order to obtain sufficient cell numbers for therapeutic purposes.

Providing safe and regulated cell therapy products to patients requires adherence to good manufacturing practices (GMP), and GMP guidelines should be adhered to throughout the process of isolating, expanding and differentiating ASCs (Giancola et al., 2012). The numerous reagents used to isolate and expand ASCs for research purposes are animal-derived or are not of clinical-grade; therefore, these need to be replaced with more suitable alternatives according to GMP standards (Halme and Kessler, 2006; Riis et al., 2015). We review the choice of serum supplementation that can be used for ASC expansion in lieu of fetal bovine serum (FBS), and describe their effects *in vitro* and *in vivo* as reported in the literature.

## International society of cellular therapy (ISCT) and international fat applied technology society (IFATS) guidelines and techniques used to assess adipose-derived stromal cell characteristics

A set of minimal criteria and guidelines have been recommended by the International Society of Cellular Therapy (ISCT) and International Fat Applied Technology Society (IFATS) for the characterization of ASCs (Dominici et al., 2006; Bourin et al., 2013). These criteria include the ability of the ASCs to adhere to plastic, their surface marker profile and their trilineage differentiation potential. The latest position paper describes viability and proliferation as additional measurements to the original characterization criteria. Furthermore, experimental methods and assays have been defined to measure the characterization criteria (Bourin et al., 2013). These criteria have been shown to be affected by numerous factors such as the liposuction technique, the SVF isolation technique and the media and supplementation used during the expansion process (Koellensperger et al., 2014; Bajek et al., 2015; Busser et al., 2015). According to the ISCT and IFATS guidelines, it is recommended and accepted research practice to confirm adherence to the above guidelines for each isolation and culture condition in order to classify the resulting cell population as ASCs.

### Techniques and methods used to characterize ASCs

#### Morphology and adherence

Once seeded, adherent ASCs display a distinct morphology, which can be described as thin, elongated and spindle-shaped. The morphological assessment of ASCs is usually preformed using light microscopy (Trojahn Kølle et al., 2013).

#### Proliferation

The ISCT and IFATS guidelines have recommended that the proliferation and frequency of progenitor ACSs are measured by a fibroblastoid colony-forming unit assay (Bourin et al., 2013). Other techniques used in the studies cited in this review make use of counting viable cells or measuring the proliferative capacity of ASCs using immunohistochemistry. Counting methods include (1) counting the cells using a viability dye and a hemocytometer, (2) counting the cells using either counting beads or staining techniques and flow cytometric analysis, and (3) using colorimetric assays that measure viable cells in a spectrophotometer (Gharibi and Hughes, 2012; Trojahn Kølle et al., 2013; Bogdanova et al., 2014; Atashi et al., 2015; Johal et al., 2015; Oikonomopoulos et al., 2015).

#### Immunophenotype

The ISCT and IFATS guidelines have listed the expression of multiple surface markers and their expected percentages as a firm requirement in their position statement. They have also recommended that surface marker expression should be measured by multi-color antibody staining (Bourin et al., 2013). Studies in this review made use of flow cytometric analysis to measure surface marker expression (Müller et al., 2006; Lindroos et al., 2009; Chieregato et al., 2011; Josh et al., 2012; Trojahn Kølle et al., 2013; Bogdanova et al., 2014; Patrikoski et al., 2014).

#### Trilineage differentiation

Differentiation into adipose, bone, and cartilage has traditionally been measured using histochemical staining techniques visualized under microscopy; however, the ISCT and IFATS guidelines have recommended that qualitative assessments should be replaced or supplemented with quantitative approaches such as measuring lineage-specific mRNA expression using reverse transcription quantitative polymerase chain reaction (RT-qPCR) techniques (Bourin et al., 2013). The techniques that have been used to measure differentiation capacity, described in the studies cited in this review, have varied from histochemical staining, to conventional PCR and RT-qPCR. Histochemical staining techniques include staining the cells with either (1) oil red O and nile red for adipogenesis; (2) Alizarin red S, alkaline phosphatase and von Kossa for osteogenesis; or (3) Alcian blue and safranin for chondrogenesis (Müller et al., 2006; Kocaoemer et al., 2007; Hebert et al., 2010; Rajala et al., 2010; Koellensperger et al., 2014; Oikonomopoulos et al., 2015; Riis et al., 2016).

## Serum supplementation

### Fetal bovine serum

FBS is the traditional serum supplement used for cell culture. FBS contains growth factors (GFs) and other elements essential for ASC attachment, expansion, maintenance, and proliferation *in vitro* (Lennon et al., 1995, 1996; Zuk et al., 2001; van der Valk et al., 2010). FBS is prone to batch-to-batch variation, xenoimmunization, and possible contamination with mycoplasma, viruses, endotoxins, and prions (van der Valk et al., 2004, 2010; Chieregato et al., 2011; Kyllonen et al., 2013; Jin et al., 2015). The source and quality of FBS may affect the proliferation and differentiation of ASCs, and routine screening for mycoplasma, endotoxins and viruses has become important (Naaijkens et al., 2012). These factors may affect experimental outcomes and render the cell product unsafe for clinical use (Zuk et al., 2001; van der Valk et al., 2004; Witzeneder et al., 2013).

ASCs are immunoprivileged, lacking the expression of the major histocompatibility complex class II as well as T and B cell costimulatory molecules (CD80, CD86, and CD40). The *in vitro* immunogenicity and immunosuppressive properties of ASCs are usually measured by co-culturing the ASCs with peripheral blood mononuclear cells in mixed lymphocyte reactions and measuring the T-cell proliferative response (McIntosh et al., 2006; Patrikoski et al., 2014). ASCs demonstrate immunomodulatory and immunosuppressive properties, as demonstrated by their ability to regulate T-cell function and modulate cytokine secretion *in vitro* and *in vivo* (Leto Barone et al., 2013; Roemeling-van Rhijn et al., 2013; Patrikoski et al., 2014). These properties arise from the low immunogenicity of ASCs. The majority of ASC and other mesenchymal stem cell (MSC) clinical trials (phase I, II, and III) use FBS supplemented media, and it has been reported that immunogenic effects are elicited by components of FBS in human subjects (Sundin et al., 2007; Riis et al., 2015). For example, a clinical trial using bone marrow-derived MSCs (BM-MSCs) expanded in FBS found antibodies against components of FBS (Horwitz et al., 2002). Immune responses to FBS such as Arthus and anaphylactic reactions have been reported in clinical trials, where patients were treated with dendritic cells and lymphocytes exposed to FBS (Selvaggi et al., 1997; Mackensen et al., 2000). In contrast, a meta-analysis of MSC clinical trials found that over 75% of experiments used FBS in their cell expansion protocols and only one study monitored and demonstrated adverse reactions to FBS (Lalu et al., 2012). *In vivo* studies examining the immune response of mice to ASCs showed preserved immunosuppression and immunomodulation, low immunogenicity, and no reaction to FBS (Cho et al., 2009; González et al., 2009). Although ASCs are being extensively tested in clinical trials, their definitive use as a therapeutic agent remains to be established. This is further compounded by the use of preclinical models that may not be biologically relevant (Monsarrat et al., 2016). Furthermore, FBS may be less immunogenic in mice and other animal models than in humans. Finally, the immune response elicited by FBS (Selvaggi et al., 1997; Mackensen et al., 2000; Horwitz et al., 2002) could conceivably influence the rejection of transplanted cells in cell-based therapy.

### Serum-free alternatives

The unknown and undefined composition of FBS is a major drawback. A preferable alternative would be a chemically-defined medium with a known composition such as commercially available serum-free (SF) or XF media (Usta et al., 2014). These serum-free media are erroneously presumed to be devoid of any animal products since the terms SF and XF are often used interchangeably. However, SF media are usually supplemented with animal-derived or human serum albumin and GFs in undefined amounts (Patrikoski et al., 2013). Xeno-free media, on the other hand, are chemically-defined media containing well-defined components at specific concentrations (Usta et al., 2014).

### Growth factors

Another alternative to serum is the addition of GFs to culture medium, either in isolation, or as a cocktail. These GFs can be synthetic, animal-derived, or human-derived. Replacement with synthetic GFs is preferable due their higher quality and as a result of standardization between batches, which may not be possible for animal- or human-derived GFs. Commonly used GFs are fibroblast growth factor, epidermal growth factor and platelet-derived growth factor (PDGF; Baer and Geiger, 2012; Ahearne et al., 2014). The addition of GFs has been linked to an increase in proliferation (Hebert et al., 2010; Gharibi and Hughes, 2012). An improved adipogenic differentiation potential has previously been reported in ASCs expanded in GF supplemented medium (Hebert et al., 2010). However, another study observed a negative effect on adipogenic and osteogenic differentiation in long term cultured ASCs (Gharibi and Hughes, 2012).

### Serum albumin

Serum albumin is an abundant plasma protein and can be isolated from humans and animals. Often SF media are supplemented with serum albumin. Studies comparing human serum albumin for ASC media supplementation have found improved proliferation, a smaller spindle-like morphology and preserved differentiation into adipose, bone and cartilage (Rajala et al., 2010; Johal et al., 2015).

### Chemically-defined XF medium

Xeno-free medium has been recommended as a replacement for FBS and serum, as it contains the necessary components for ASC expansion, does not involve donor or batch-to-batch variation, is GMP compliant and has minimal immunogenicity and favorable immunosuppression (van der Valk et al., 2004, 2010; Usta et al., 2014). When compared to FBS, the use of XF medium for the expansion of ASCs has led to better morphological quality, increased proliferation, a comparable immunophenotype and differentiation into adipose, bone and cartilage (Lindroos et al., 2009; Patrikoski et al., 2013; Oikonomopoulos et al., 2015). The use of XF media in ASC expansion results in ASCs losing their ability to adhere to plastic (Kyllonen et al., 2013; Patrikoski et al., 2013; Oikonomopoulos et al., 2015). Additional coating agents are needed to maintain the inherent characteristic of plastic-adherence associated with ASCs. Commercially available XF medium is expensive and preparing in-house XF medium can be time-consuming and may increase the risk of batch-to-batch variation (Lund et al., 2009; Baer et al., 2010; Rajala et al., 2010; Yang et al., 2012; Kyllonen et al., 2013; Patrikoski et al., 2013; Oikonomopoulos et al., 2015).

### Human alternatives

Human alternatives can replace FBS and SF/XF supplemented media and can create a culture environment that more accurately resembles the human environment (Azouna et al., 2012; Koellensperger et al., 2014). Furthermore, the use of autologous products (derived from the same individual) obviates the need for testing for infectious and other disease causing agents.

#### Human serum

**Figure 1:**
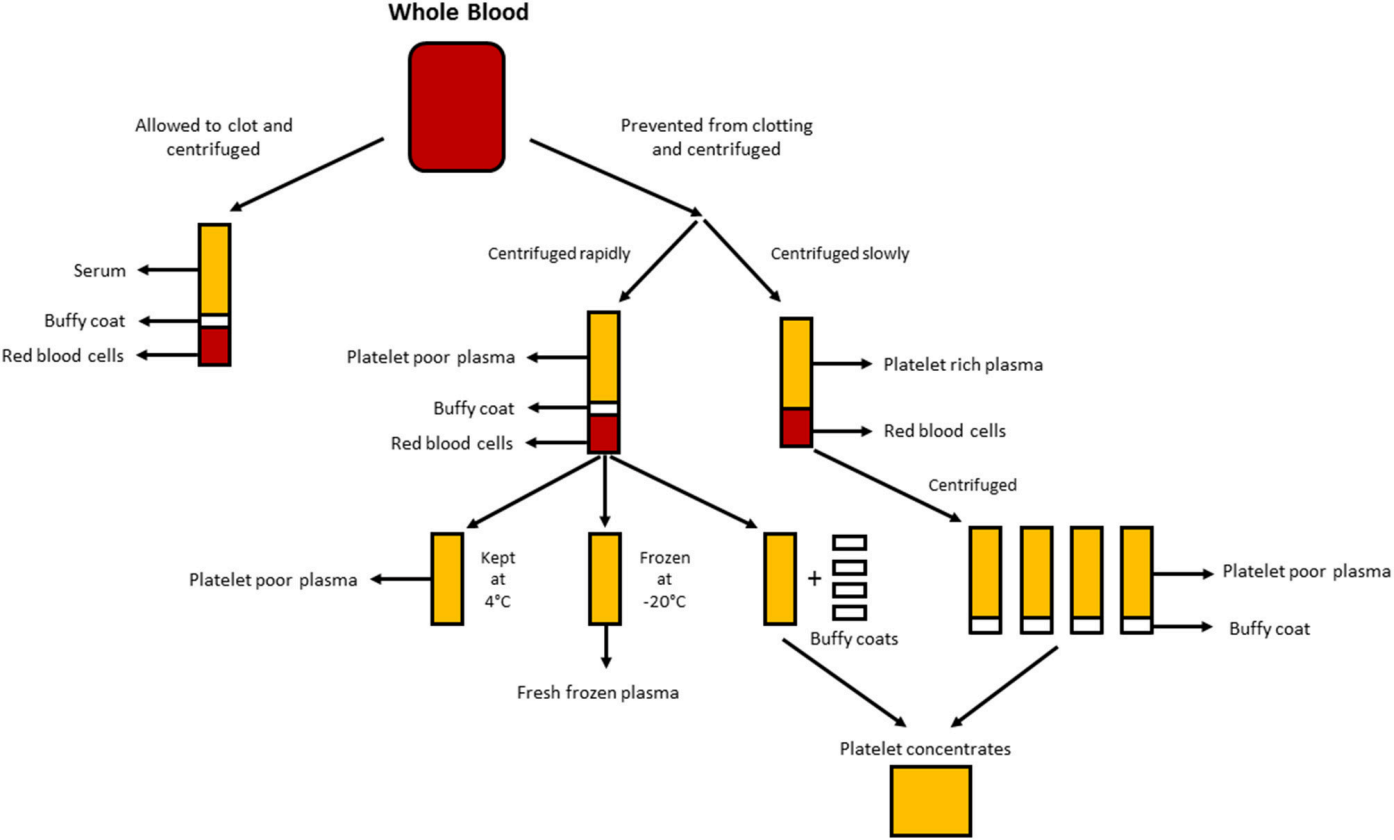
**Production of the different human alternatives**. Serum is produced when whole blood is allowed to clot and centrifuged to pellet red and white blood cells, and platelets. Plasma is produced by the prevention of clotting followed by centrifugation. Depending on the centrifugation speed, either platelet poor plasma (PPP; rapid centrifugation) or platelet rich plasma (PRP; slower centrifugation) is produced. If the PPP is stored at −18°C it is known as fresh frozen plasma. Platelet concentrates can be produced either by taking the platelet poor plasma and 4 buffy coats and pooling them together or centrifuging multiple PRP's and pooling the platelet pellets (suspended in a small amount of plasma) together.

After whole blood has been allowed to clot in the absence of an anticoagulant and has been centrifuged, serum is the resulting liquid portion that does not contain platelets, white blood cells or red blood cells (Figure 1; Stedman, 2006). Human serum (HS) can either be autologous (donor and recipient are the same individual) or allogeneic (derived from individuals who are different from the recipient). Both autologous and allogeneic HS are superior to FBS (Stute et al., 2004; Bieback et al., 2009, 2012; Bernardo et al., 2011; Kyllonen et al., 2013; Patrikoski et al., 2013). ASCs expanded in HS have greater transcriptome stability than those expanded in FBS, whereas genes responsible for cell cycle prolongation, differentiation and extracellular matrix and prostaglandin synthesis are upregulated and overexpressed in FBS when compared with HS using microarray analysis (Shahdadfar et al., 2005). ASCs expanded in FBS reached senescence sooner and displayed telomere shortening when compared to ASCs expanded in HS (Shahdadfar et al., 2005). The choice of HS seems to have little effect on the immunomodulatory properties of ASCs. ASCs expanded in either allogeneic HS or FBS containing media had low immunogenicity and resulted in immunosuppression (Patrikoski et al., 2014). ASCs expanded in either autologous or allogeneic HS display greater proliferation and an indistinguishable immunophenotype when compared to ASCs expanded in FBS (Josh et al., 2012; Bogdanova et al., 2014). ASCs expanded in allogeneic HS have been differentiated into adipose, bone and cartilage, although the upregulation of chondrogenic and osteogenic genes was favored compared to FBS (Josh et al., 2012). ASCs expanded in autologous HS have been differentiated into adipose and cartilage; however, the ability to differentiate into bone was less-favored (Bogdanova et al., 2014). Autologous HS may provide ASCs with better proliferation and genomic stability as determined by microarray analysis when compared to allogeneic HS (Shahdadfar et al., 2005; Bieback et al., 2009; Bernardo et al., 2011). ASCs expanded in allogeneic HS entered growth arrest and underwent cell death (Shahdadfar et al., 2005; Lindroos et al., 2009), which limits the potential advantages of allogeneic HS. While autologous HS might be ideal, its availability is limited and there may be significant variation between patients in the ability of their own serum to support growth of their own cells (Lange et al., 2007). Alternatively, allogeneic HS can be pooled, yielding larger quantities for laboratory experimentation and can undergo rigorous quality testing by a blood bank (e.g., testing for the absence of infectious agents and contamination with other blood cells) prior to use in humans (Bieback et al., 2009).

#### Plasma

Plasma is the non-cellular liquid portion of blood that contains water, electrolytes and proteins (clotting factors, fibrinogen, and anticoagulants). Platelet poor plasma (PPP), fresh frozen plasma (FFP), and platelet rich plasma (PRP; Figure 1) can be obtained from whole blood by centrifugation at different speeds, and subsequent storage at different temperatures. GF secretion can be enhanced by activating the platelets in the whole blood with thrombin prior to centrifugation (Doucet et al., 2005; Kocaoemer et al., 2007), thereby enhancing the activity of the plasma products.

#### Platelet poor plasma

PPP is almost free of platelets and is produced from whole blood by the addition of an anticoagulant during the collection process, after which the plasma is separated using rapid centrifugation (Figure 1; Koellensperger et al., 2006). This allows the platelets and red blood cells to be pelleted. The resulting PPP is stored at 4°C and is referred to as fresh plasma. PDGF is secreted by the aggregating platelets; however, negligible PDGF is released in PPP as a result of the small number of residual platelets. GFs may thus need to be added to PPP when used in media as is the case when using SF medium (Müller et al., 2006; Gottipamula et al., 2013). Using PPP without the addition of GFs has resulted in lower proliferation rates and a smaller increase in DNA synthesis as measured using thymidine incorporation, when compared with HS and FBS (Vogel et al., 1980; Koellensperger et al., 2006). PPP with added GFs resulted in increased proliferation rates compared to HS (Koellensperger et al., 2006); however, these differences could have arisen from different production protocols of PPP and the addition of varying levels of GFs to each of the PPP preparations in this study. Expansion of ASCs in PPP results in improved proliferation when compared to FBS, and has osteogenic differentiation which is comparable to that of ASCs expanded in HS (Koellensperger et al., 2014). Chondrogenic differentiation was decreased in ASCs expanded in PPP when compared to ASCs expanded in PRP (Koellensperger et al., 2014).

#### Fresh frozen plasma

FFP is obtained in the same manner as PPP, but it is frozen directly after separation at −18°C (O'Shaughnessy et al., 2004; Liumbruno et al., 2009). FFP has been used in the expansion of BM-MSCs with positive results. These results include better proliferation, immunosuppressive activity, and differentiation into adipocytes and osteocytes; and an immunophenotype and morphology that is comparable to cells expanded in FBS (Müller et al., 2006; Mannello and Tonti, 2007). However, the use of FFP as a serum substitute in ASC expansion requires further investigation.

#### Platelet rich plasma

PRP is the portion of blood that is enriched in platelets. PRP is produced by separating plasma from red blood cells at slower centrifugation speeds, which prevents the pelleting of the platelets (Figure 1). ASCs expanded in PRP maintain a classic immunophenotype and morphology, and PRP increases proliferation when compared to FBS (Kocaoemer et al., 2007; Chieregato et al., 2011; Atashi et al., 2015). ASCs expanded in PRP have improved differentiation efficiency toward adipogenic and osteogenic lineages, while having comparable efficiency for chondrogenic differentiation, when compared to ASCs expanded in FBS (Kocaoemer et al., 2007; Chieregato et al., 2011). When compared, HS was found to be slightly better than PRP in terms of differentiation and proliferation of ASCs (Kocaoemer et al., 2007; Chieregato et al., 2011). PRP is a poorly defined culture medium supplement due to its high biological variability and complicated extraction procedure, in which purifying the platelet factor-rich supernatant from plasma membranes can be difficult. The use of PRP is limited by the large quantities of whole blood needed to yield enough PRP for experimentation (Chieregato et al., 2011).

#### Platelet lysate

Human platelet lysate (HPL) contains platelet GFs which are obtained by lysing platelets concentrated in a small volume of plasma (platelet concentrates; Figure 1) by temperature shock. HPL contains a higher concentration of GFs than other serum substitutes including human PRP and FBS (Doucet et al., 2005; Bernardo et al., 2006, 2011; Bieback et al., 2009; Schallmoser et al., 2010). HPL can easily be obtained and produced from apheresis products and buffy coats, and can be resuspended in either PRP or an additive solution (Schallmoser and Strunk, 2013; Iudicone et al., 2014). HPL is produced by freezing platelets at between −30 and −80°C for 24 h, followed by a thawing and centrifugation step. The repeated freeze, thaw and centrifuge cycles allow for the release of GFs and the removal of platelet bodies (Bernardo et al., 2006; Schallmoser et al., 2007). Another benefit of HPL supplementation is that platelets can be used after the 4–5 day expiry date of banked blood (Bieback et al., 2009). HPL is a better alternative than autologous and allogeneic HS, as ASCs expanded in HPL maintain their classic immunophenotype, differentiation, clonogenic efficiency, cell purity, and cell viability (Trojahn Kølle et al., 2013; Riis et al., 2016). HPL also supports long-term expansion without compromising the immunomodulatory properties of ASCs, as measured by flow cytometric analysis (Bieback et al., 2009). Expansion in HPL results in a shorter population doubling time, reducing the time required for cell expansion and lowering the threat of senescence and transformation (Doucet et al., 2005; Shahdadfar et al., 2005; Bernardo et al., 2006, 2011; Azouna et al., 2012). The bio-safety of HPL has been assessed using array comparative genomic hybridization and high sensitivity spectral karyotyping, where it was found that ASCs expanded in HPL had no chromosomal aberrations (Crespo-diaz et al., 2011; Trojahn Kølle et al., 2013). Classic ASC morphology (thin, smaller, elongated, and spindle-shaped) is maintained in HS and HPL, whereas ASCs expanded in FBS are larger and less spindle-shaped (Trojahn Kølle et al., 2013). Although this may indicate that both HS and HPL select for primitive/immature ASCs (Doucet et al., 2005; Bieback et al., 2009), it also suggests that cells grown in FBS have reduced proliferation and progress more rapidly toward senescence. HPL varies between individuals (Bernardo et al., 2006; Crespo-diaz et al., 2011), and batch-to-batch variation is reduced when HPL is pooled (Schallmoser et al., 2007; Trojahn Kølle et al., 2013). Moreover, by pooling many donors, a large quantity can be obtained for supplementation, which makes HPL preferable to PRP (Kocaoemer et al., 2007; Bieback et al., 2009; Chieregato et al., 2011).

## Conclusion

According to Riis et al. of all the registered clinical trials using expanded ASCs that have listed their expansion conditions, the majority make use of FBS, three trials use autologous HS, one trial uses PRP, and one trial uses HPL (Riis et al., 2015). These statistics are alarming, as FBS has the potential to transmit zoonotic diseases following cell transplantation, and immune reactions against FBS components have been reported (Selvaggi et al., 1997; Mackensen et al., 2000). FBS is a non-GMP compliant product, as it affects the safety and efficacy of the ASC therapeutic, and thus needs to be replaced (van der Valk et al., 2004; Kyllonen et al., 2013; Witzeneder et al., 2013). This has been remedied by replacing FBS with chemically-defined human derived alternatives. Changing from FBS to human alternatives or XF/SF media in regenerative medicine has the important advantage that the ASCs proliferate much faster in the latter, resulting in a greater number of cells for transplantation in a shorter time. However, the relative superiority of different culture media is still widely debated. Studies comparing more than one culture medium have reported varying results (Lange et al., 2007; Bernardo et al., 2011; Koellensperger et al., 2014; Riis et al., 2016). Koellensperger et al. compared the trilineage differentiation of ASCs expanded in FBS, PRP, PPP, and HS (Koellensperger et al., 2014). Their results revealed that each culture medium allowed differentiation into one or more lineages, but never into all three lineages. When XF media, FBS and HPL supplemented media were compared, Riis et al. found that certain subpopulations expressed specific surface markers depending on the culture medium utilized (Riis et al., 2016). Studies comparing the immunophenotype of ASCs expanded in FBS and the other culture media, found little to no difference in cell surface marker expression, irrespective of the markers studied (Table 1). Seeding density, oxygen tension, confluency, dissociation, and the choice of basal media may also influence experimental outcomes (Sotiropoulou et al., 2006; Freshney, 2010; Bourin et al., 2013; Inamdar and Inamdar, 2013; Feng et al., 2014; Riis et al., 2015). The choice of culture medium depends on the downstream application of these cells (administration of differentiated or non-differentiated ASCs) and the condition being treated. Additionally, the immunogenicity of the culture medium used to expand the cells prior to clinical application should be considered as a parameter that might influence the clinical outcome. Most studies comparing different culture media used the criteria specified by the ISCT and IFATS to validate the use of an alternative to FBS. Most of these studies examine ASC morphology, proliferation, immunophenotype, and the ability of these cells to differentiate along osteogenic, chondrogenic, and adipogenic lineages in different culture media. Few studies have explored other aspects of ASCs, such as senescence, genetic stability, transcriptome, proteome, immunogenicity, cytokine secretion, and cell cycle (Shahdadfar et al., 2005; Bieback et al., 2012). While the ISCT and IFATS criteria were an attempt to unify the field in terms of standard operating procedures (Dominici et al., 2006; Bourin et al., 2013), no consensus exists around which properties of ASCs are relevant for clinical trials, making the comparison of different culture media virtually impossible. While these criteria provide measurable outcomes for easy comparison, changing components used for the expansion of ASCs may have different effects on the safety, efficacy and reproducibility of ASC end products. Examining changes in the transcriptome, proteome, and secretome of ASCs expanded in various culture media is important, as is the use of cells expanded under varying conditions in appropriate preclinical models.

**Table 1:** **The effects of the different media supplements on ASCs *in vitro***.

**Serum alternative**	**Morphology**	**Immunophenotype**	**Differentiation**	**Proliferation**	**References**
FBS	Spindle-shaped or spread out, larger in size	Positive expression for CD73, 90, 105, and negative expression for CD34 and 45	Ability to differentiate into bone, fat, cartilage. Better differentiation into adipose	Slower proliferation when compared to other alternatives	Dominici et al., 2006; Bourin et al., 2013
HS: autologous	Smaller size and tighter spindle-shape	Comparable expression to that of FBS	Differentiation into adipose and cartilage, conflicting evidence on osteogenesis	Improved proliferation when compared to FBS	Josh et al., 2012; Bogdanova et al., 2014
		Negative: CD31, CD34, and CD45			
		Positive: CD73, CD90, and CD105			
HS: allogeneic	Smaller size and tighter spindle-shape	Comparable expression to that of FBS	Differentiation into adipose and cartilage, conflicting evidence on osteogenesis	Improved proliferation when compared to FBS	Josh et al., 2012; Bogdanova et al., 2014
		Negative: CD34, CD14, CD19, CD45, and HLA-DR			
		Positive: CD29, CD44, CD73, CD90, and CD105			
PPP	Spindle-shaped or spread out, larger in size	Comparable expression to that of FBS	Differentiation into adipogenesis comparable, better osteogenesis, less chondrogenesis	Improved proliferation when GFs added, lower proliferation rate without GFs added when compared to FBS	Koellensperger et al., 2006, 2014
		Negative: CD31, CD34, CD44, CD45, and CD106			
		Positive: CD13, CD29, CD49a, CD63, CE73, CD90, CD105, and CD166			
PRP	Smaller size and tighter spindle-shape	Comparable expression to that of FBS	Preserved trilineage differentiation	Improved proliferation when compared to FBS	Kocaoemer et al., 2007; Chieregato et al., 2011
		Negative: CD133, CD31, CD34, CD45, CD144, and CD117			
		Positive: CD29, CD73, CD105, CD90 and CD 44			
HPL	Smaller size and tighter spindle-shape	Comparable expression to that of FBS	Trilineage differentiation retained	Improved proliferation when compared to FBS	Trojahn Kølle et al., 2013
		Negative: CD31, CD14, CD20, CD34, and CD45			
		Positive: CD90, CD73, and CD105			
XF	Smaller size and tighter spindle-shape	Comparable expression to that of FBS	Trilineage differentiation retained	Improved proliferation when compared to FBS	Lindroos et al., 2009; Patrikoski et al., 2013
		Negative: CD11a, CD14, CD19, CD80, CD86, CD31, CD34, CD45, CD106, CD146, and HLA-DR			
		Positive: CD29, CD10, CD13, CD73, CD90, CD105			
		Variation in CD54, 49d, CD9, and CD166			
Serum albumin	Better morphological quality	Comparable expression to that of FBS	No data available	Improved proliferation	Trivedi et al., 2008
		Negative: CD34 and CD45			
		Positive: CD73 and CD90			
GF supplementation	Better morphological quality	Comparable expression to that of FBS	Contradictory data, either improved adipogenic capacity when compared to FBS or less adipogenic and osteogenic differentiation	Improved proliferation	Hebert et al., 2010; Gharibi and Hughes, 2012; Johal et al., 2015
		Negative: CD34, CD45			
		Positive: CD105, CD90, CD44, CD71, and CD146			

## Author contributions

CD conceptualized and drafted the review, and approved the final manuscript. MP assisted in the conceptualization of the review, revised, and approved the final manuscript. MSP assisted in the conceptualization of the review, edited, and approved the final manuscript, and raised the funding for the projects concerned. All authors have read and approved the final manuscript.

## Funding

This research and the publication thereof is the result of funding provided by the Medical Research Council of South Africa in terms of (a) the MRC's Flagships Awards Project SAMRC-RFA-UFSP-01-2013/STEM CELLS as well as (b) the Extramural Unit for Stem Cell Research and Therapy. The National Research Foundation of South Africa also provided funding.

### Conflict of interest statement

The authors declare that the research was conducted in the absence of any commercial or financial relationships that could be construed as a potential conflict of interest.
